# Depot-dependent effects of subclinical ketosis on visceral and subcutaneous adipose tissue transcriptional cellular diversity in dairy cows

**DOI:** 10.1186/s40104-025-01265-y

**Published:** 2025-09-21

**Authors:** Hunter Ford, Clarissa Strieder-Barboza

**Affiliations:** 1 Department of Veterinary Sciences, Davis College of Agricultural Sciences and Natural Resources, Texas Tech University, Lubbock, TX USA; 2 School of Veterinary Medicine, Texas Tech University, Amarillo, TX USA

**Keywords:** Adipose, Dairy, Depot, Ketosis, Single-nuclei

## Abstract

**Background:**

Adipose tissue plays a central role in regulating whole-body metabolic health, facilitated by the variety of cell types and their wide-ranging functions. In addition, depot-specific differences in adipose tissue have been shown to play important roles in different disease states including obesity, diabetes, and metabolic dysfunction in human and animal models. For early postpartum dairy cattle, metabolic dysfunction, triggered by a negative energy balance, is often manifested as subclinical ketosis (SCK). However, the role that subcutaneous (SAT) and visceral (VAT) adipose tissue depots, and their diverse cellular compositions, play in the response to subclinical ketosis conditions is unclear.

**Results:**

Flank SAT and omental VAT were collected via laparotomy from five non-ketotic (NK; BHB ≤ 0.8 mmol/L) and five subclinical ketosis (SCK; 1.4 mmol/L < BHB ≤ 2.6 mmol/L) multiparous cows during early lactation. Following collection, nuclei were isolated from the tissue and subjected to single-nuclei RNA sequencing in order to investigate the transcriptional cellular heterogeneity. Distinct clusters of adipocytes (AD), adipose stem/progenitor cells (ASPC), immune cells (IMC), endothelial cells (EC), and pericyte/smooth muscle cells (PE/SMC) were identified in both adipose depots, with a greater abundance of ASPC in SAT compared to VAT. In addition, we identified a VAT-specific AD subtype characterized by higher expression of progenitor-like marker genes. While the abundance of none of the identified cell subtypes were different between SCK and NK, underlying transcriptional changes provided insight into potential effects of SCK. In general, SCK was associated with pro-lipogenic, anti-inflammatory, and pro-angiogenic transcriptional changes, possibly indicating a greater capacity for homeostatic responsiveness in SAT under conditions of enhanced negative energy balance. In contrast, SCK appeared to promote transcriptional changes indicative of impaired adipogenesis, impaired angiogenesis, and increased inflammation in VAT.

**Conclusions:**

Uniquely, our study presents novel insight into the cellular heterogeneity of adipose tissue in dairy cattle with subclinical ketosis. Furthering our understanding of the role of adipose tissue in response to this form of metabolic challenge has the potential to enhance efforts aimed at limiting the incidence and impact of subclinical ketosis and improving the health and productivity of dairy cattle.

**Supplementary Information:**

The online version contains supplementary material available at 10.1186/s40104-025-01265-y.

## Introduction

In dairy cattle, adipose tissue plays a critical role in supporting the metabolic adaptation from gestation to lactation. While energy demands increase due to the onset of lactation, energy availability is reduced due to decreased feed intake [1, 2], resulting in a negative energy balance. Adipose tissue mobilization via lipolysis serves as an alternative energy source when the majority of glucose is being utilized in the mammary gland [2, 3]. While this process is physiological and necessary to support early lactation, excess circulating lipids, in the form of non-esterified fatty acids (NEFA), can lead to increased production of ketone bodies. Ketones are important alternative energy sources; however, subclinical ketosis, mainly during the first week of lactation [4], is directly associated with reduced productive and reproductive performance, and increased risk of disease in dairy cattle [5]. Unlike clinical ketosis, subclinical ketosis (SCK) is characterized by the absence of clinical signs, with blood β-hydroxybutyrate concentrations of 1.2 to 2.9 mmol/L [6–8]. Furthermore, with an incidence of 40%–60% [9] and an estimated cost of $256 per case in multiparous cows [10], SCK presents a significant economic burden for the dairy industry. However, recent investigations have questioned whether SCK is a true disease or a transitory metabolic state reflective of an adaptation to the negative energy balance in early lactation, particularly for high-producing cows [4, 5]. As such, a more comprehensive understanding of the relationship between adipose tissue function and SCK is necessary.

Recent advances in transcriptomic techniques, particularly single-nuclei RNA sequencing (sn-RNASeq), have revealed the cellular heterogeneity of adipose tissue comprised of mature adipocytes, adipose stromal and progenitor cells, vascular cells, and immune cells [11, 12]. This complex milieu of cell types contributes to adipose tissue’s role as a regulator of whole-body energy homeostasis. However, not all adipose tissue is the same, with notable differences both in functional and cellular heterogeneity between subcutaneous (SAT) and visceral (VAT) adipose tissue, the two main adipose depots in dairy cattle. While SAT has a greater capacity for adipogenesis, evidenced by increased proliferation of adipocyte progenitor cells and lipid accumulation, VAT appears to be more immunomodulatory, with a greater abundance of immune cells and increased expression of inflammatory and immune-related genes [13]. In terms of cellular heterogeneity, Michelotti et al. [11], noted increased abundance of endothelial cells and adipose stromal and progenitor cells in SAT compared to VAT, with an increased abundance of macrophages and natural killer/T cells in VAT compared to SAT. In addition, greater activation of immune and inflammatory pathways supports distinct depot-specific functional differences in these adipose tissue depots of dairy cattle. However, the relationship between these depot differences in cellular composition and function and the early lactation metabolic adaptations, including their link to a ketotic status in periparturient dairy cattle remain poorly understood.

Given the importance of adipose tissue in the metabolic response of dairy cattle to the energy demands of early lactation, there is a need to better define this tissue at the cellular and molecular levels to advance the development of targeted approaches to improve dairy cow health and productivity. Thus, the objective of this study was to elucidate the cellular heterogeneity of SAT and VAT in early postpartum dairy cattle with and without SCK utilizing a sn-RNASeq approach.

## Materials and methods

### Animals and adipose tissue sample collection

All procedures performed in this investigation were approved by the Institutional Animal Care and Use Committee (IACUC) of Texas Tech University (protocol # 21024-04). Details regarding the selection of the ten dairy cattle utilized in this study as well as the number of lactations, body condition scores (BCS), days in milk (DIM), serum metabolic parameters, and description of the AT sampling are available in previous publications [14, 15]. Briefly, five cows were assigned to the non-ketotic group (NK; *n* = 5; BHB ≤ 0.8 mmol/L; 3.2 ± 1.6 lactations; 7.4 ± 1.8 DIM; 3.7 ± 0.2 BCS) and five cows were assigned to the subclinical ketosis group (SCK; *n* = 5; BHB > 1.4 and ≤ 2.6 mmol/L; 3.2 ± 1.2 lactations; 7.8 ± 2.0 DIM; 3.6 ± 0.4 BCS). A summary of descriptive statistics is provided in Table 1. Adipose tissue samples of flank SAT and omental VAT were collected via laparotomy from each cow, as described in previous publications [14, 15]. Briefly, laparotomies were performed on one control and one SCK animal each day. A large area over the right paralumbar fossa, bordered dorsally by the transverse processes of the Lumbar vertebrae, caudally by the tuber coxae, and cranially by the last rib, was clipped to remove hair. The surgical site was cleaned with water and soap, followed by two rounds of antisepsis using povidone-iodine and 70% ethanol. Local anesthesia was administered using 40–50 mL of 2% lidocaine hydrochloride (CAT# 510213, VetOne) in an inverted L-block, with infiltration performed at least 7 cm from the intended incision site. A 10–12 cm skin incision was made at the center of the paralumbar fossa using a scalpel. Approximately 10 g of subcutaneous adipose tissue (SAT) was collected from beneath the skin. The incision continued through the muscle layers to reach the peritoneal cavity. Once the greater omentum was located, approximately 20 g of omental adipose tissue (VAT) was collected near the greater curvature of the abomasum. Following tissue collection, the peritoneum and transversus abdominis muscle were sutured together using a continuous suture pattern with synthetic absorbable violet coated braided polyglactin 910-USP 3 + 4 (Riverpoint Medical, Portland, OR, USA). The internal and external oblique muscles were closed together in a second layer. The skin was sutured using a Ford interlocking pattern with nonabsorbable polyamide thread, pseudomonofilament suture USP 3 (CAT# J009103, Braunamid, B. Braun Surgical, Rubi, Spain). Animals were administered flunixin meglumine (Prevail, CAT# V1502018, Distributed by MWI, Boise, ID, USA) post-surgery and were monitored for the following 3 d, with suture removal after 7–10 d. Approximately 1 g of tissue was immediately flash frozen after surgical collection and kept in liquid nitrogen until nuclei isolation.

**Table 1:** Descriptive statistics of non-ketotic (NK) and subclinical ketosis (SCK) cows

Parameter	NK	SCK	SEM	*P-*value
DIM^a^	7.4	7.8	1.02	0.59
Parity	3.2	3.2	0.80	0.99
BCS^b^	3.7	3.6	0.20	0.55
BHB^c^, mmol/L	0.72	1.58	0.05	< 0.01
NEFA^d^, mEq/dL	0.9	1.17	0.16	0.25
Glucose, mg/dL	43.6	41.8	6.42	0.83

^a^Days in milk

^b^Body condition score

^c^β-Hydroxybutyrate (cow-side measurement)

^d^Non-esterified fatty acids

### Single-nuclei isolation and processing

Approximately 500 mg of each adipose tissue sample was washed in cold, sterile, RNase-free 1X PBS (CAT# AM9625, Invitrogen) diluted in nuclease-free water (CAT# AM9932, Invitrogen), then minced using a scalpel in a petri dish on ice in 0.5 mL lysis buffer comprised of nuclease-free water with 3 mmol/L MgCl_2_ (CAT# AM9530G, Invitrogen), 10 mmol/L Tris buffer, pH 8 (CAT# AM9855G, Invitrogen), 25 mmol/L KCl (CAT# AM9640G, Invitrogen), 250 mmol/L sucrose (CAT# S0389-500G, Sigma-Aldrich), 0.1% Triton X-100 (CAT# T8787, Sigma-Aldrich), and 100 µmol/L DTT (CAT# 646563, Sigma-Aldrich). After mincing, samples were transferred and homogenized in a pre-cooled 15 mL glass dounce homogenizer (CAT# 357544, Duran Wheaton Kimble) by performing 10 strokes with Pestel A and 15 strokes with Pestel B on ice. Samples were then strained through a 40-µm filter (CAT# 22363547, ThermoFisher Scientific), pre-wet with ~200 µL of Nuclei Isolation Medium (NIM; composed of lysis buffer without Triton and DTT) into a 50-mL conical tube. The strained homogenates were then transferred into pre-cooled, sterile, RNase-free microcentrifuge tubes and centrifuged at 500 × *g* for 5 min at 4 °C. The resulting supernatant was discarded, leaving ~50 µL in the tube. The pellet was then resuspended in 500 µL of nuclei resuspension buffer containing 1% BSA (CAT# 9048-46-8, ThermoFisher Scientific), and 0.2 U/µL RNase inhibitor (CAT# 11836170001, Roche) in DPBS (CAT# 14190-144, Gibco). The samples were then centrifuged at 300 × *g* for 5 min at 4 °C, after which the supernatant was removed, leaving ~50 µL in the tube. The sample was gently resuspended in the remaining volume and 5 µL was removed for counting nuclei using Trypan Blue (CAT# 15250–061, Gibco), while the remaining sample was utilized for sequencing. Nuclei Libraries, targeting 6,000–10,000 nuclei, were prepared using the 10x Genomics Chromium Next GEM Single Cell 3′ Reagent Kits (10x Genomics). Library quality was assessed via DS 5000 HS assay kit using Tape Station 4200 (Agilent Technologies, Santa Clara, CA, United States) and quantified using Qubit dsDNA HS assay kit on Qubit Fluorometer version 2.0 (Life Technologies Inc., Carlsbad, CA, United States). Nuclei Libraries were sequenced via 150 bp paired-end sequencing using an Illumina NovaSeq 6000 (Illumina). FASTQ files were generated using the Bcl2fastq2 Conversion Software (Illumina), and the CellRanger Pipeline (10x Genomics) was utilized for alignment and generating count matrices.

### Sn-RNASeq data analysis

Sequencing, alignment and annotation of the nuclei libraries was performed as previously described by our group [11]. Analysis was performed using the Seurat (v5) package [16–20] in RStudio (v.4.3). The Seurat pipeline was run as previously described [11], with minor adjustments. Nuclei with > 15% mitochondrial genes and > 20% ribosomal genes were removed from the analysis and clustering was performed at a resolution of 1.0. Following initial quality control, one of the SAT SCK samples required removal due to a low number of nuclei. After clustering, clusters with > 90% of nuclei derived from a single sample and/or clusters that were not represented in the majority of samples were excluded from further analysis. The final dataset was composed of 9 SAT (4 SCK; 5 NK) and 10 VAT (5 SCK; 5 NK) samples, totaling 12,191 and 14,058 nuclei, respectively. Differentially expressed genes were identified in each cluster and in each group of clusters of interest as genes with Log_2_FoldChange > |0.5| and adjusted *P*-value < 0.05 in accordance with our previous single-nuclei investigation in dairy cow adipose tissue [11]. CellChat [21] (v1.6.1) analysis was performed in RStudio on the NK SAT, SCK SAT, NK VAT, and SCK VAT Seurat objects subset from the full dataset to assess predicted ligand-receptor interactions based on overexpressed genes. The ligand-receptor database for humans was used as no database specific for bovine was available. Functional analysis of differentially expressed genes for clusters and comparisons of interest was performed using clusterProfiler [22] (v4.8.3) in RStudio. Enrichment of Gene Ontology (GO) terms for Biological Process (BP), Molecular Function (MF) and Cellular Component (CC) was assessed using an adjusted *P*-value < 0.05. Following clusterProfiler functional analysis, significantly enriched GO BP terms were further analyzed using REVIGO [23] with the *Bos taurus* database to cluster enriched terms based on semantic similarity and remove obsolete GO terms.

### Statistical analysis

Significant differences in cluster and cell type abundance were assessed in SAS (v9.4, SAS Institute Inc., Cary, NC, USA) using the PROC GLM procedure with AT depot (SAT, VAT) and ketosis status (NK, SCK) as the fixed effect, with cow as the random effect. Significant differences were declared at *P* < 0.05 and tendencies at 0.05 ≤ *P* ≤ 0.1. Pearson correlation coefficients between serum BHB concentrations and cell cluster abundance were assessed using the PROC CORR procedure in SAS.

## Results

### Abundance of specific adipose tissue cell types are affected by depot, but not by ketosis

**Fig. 1:**
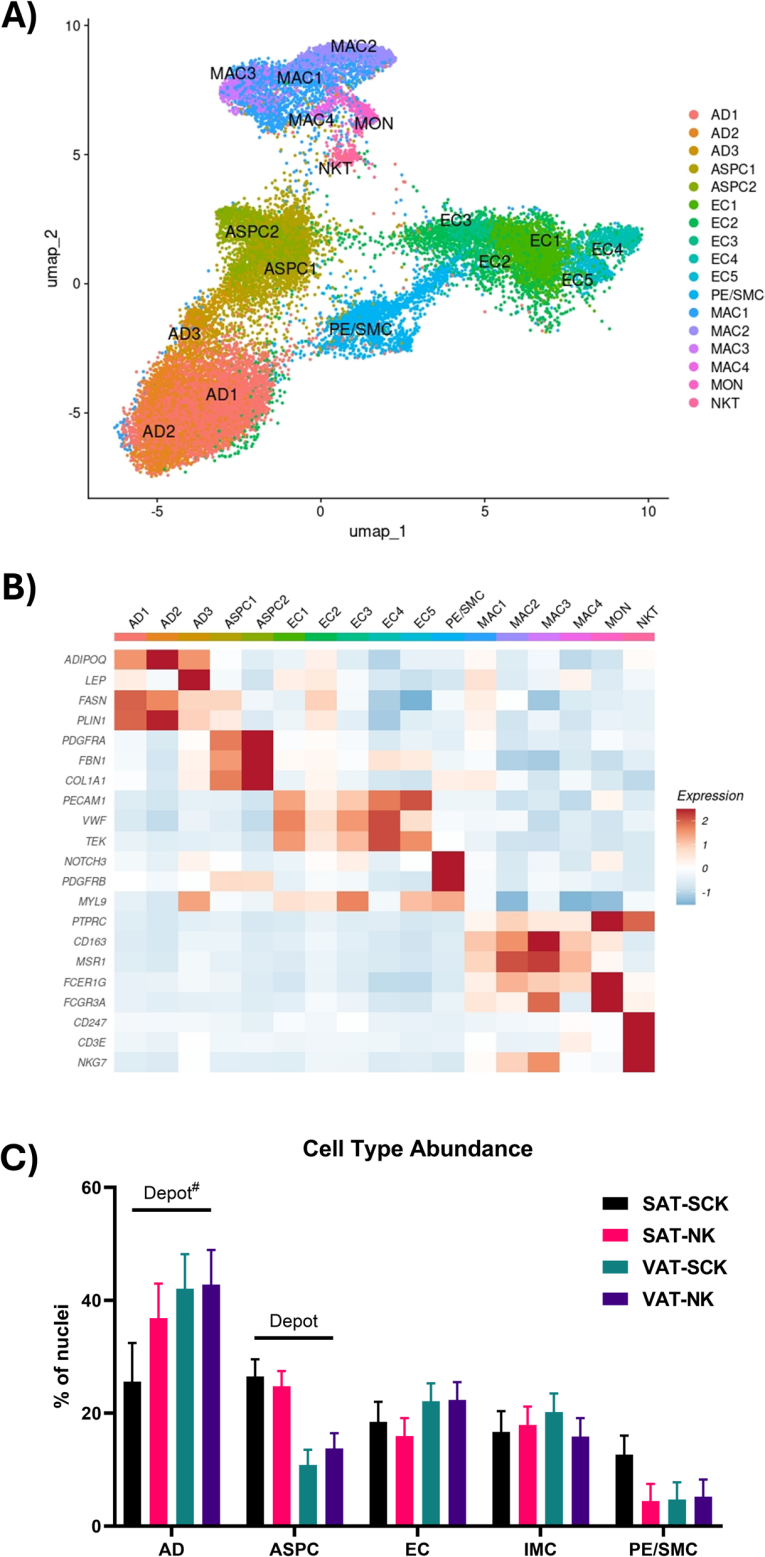
Single-nuclei RNA sequencing (sn-RNASeq) of subcutaneous (SAT) and visceral (VAT) adipose tissue from non-ketotic (NK) cows and cows with subclinical ketosis (SCK). **A** UMAP plot of nuclei in full dataset. **B** Heatmap of typical marker gene expression in each identified cell subtype. **C** Comparison of general cell type abundance in each of the four sample types. Lines over bars indicate the comparison of interest is significant (*P* < 0.05); # indicates a tendency (0.05 ≤ *P* ≤ 0.1). AD = adipocytes; ASPC = adipose stem/progenitor cells; EC = endothelial cells; PE/SMC = pericyte/smooth muscle cells; MAC = macrophages; MON = monocytes; NKT = natural killer/T-cells

We identified all the major adipose tissue cell types across SAT and VAT based on the expression of signature marker genes described in prior publications in mouse [24, 25], human [24–28] and dairy cow [11] adipose tissues (Fig. 1A and B). Identified clusters included three clusters of adipocytes (AD; *ADIPOQ*), two clusters of adipose stromal and progenitor cells (ASPC; *PDGFRA*), five clusters of endothelial cells (EC; *PECAM1*), one cluster of pericyte/smooth muscle cells (PE/SMC; *NOTCH3*), and six clusters of immune cells (IMC; *PTPRC*) comprised of four clusters of macrophages (MAC; *CD163*, *MSR1*), one cluster of monocytes (MON; *FCER1G*, *FCGR3A*) and one cluster of natural killer/T cells (NKT; *CD247*, *NKG7*). Combining the clusters based on major cell type (Fig. 1C), there was a tendency for a higher abundance of AD in VAT (*P* = 0.10) and a significantly higher abundance of ASPC in SAT (*P* < 0.01), in agreement with the cell abundances described by Michelotti et al. [11], in the adipose tissue of dairy cattle. There was no effect of SCK, or of the interaction between SCK and depot on the abundance of any major cell type.

### AD1 and AD2 represent classic adipocytes, while AD3 is a unique, VAT-specific adipocyte subtype

**Fig. 2:**
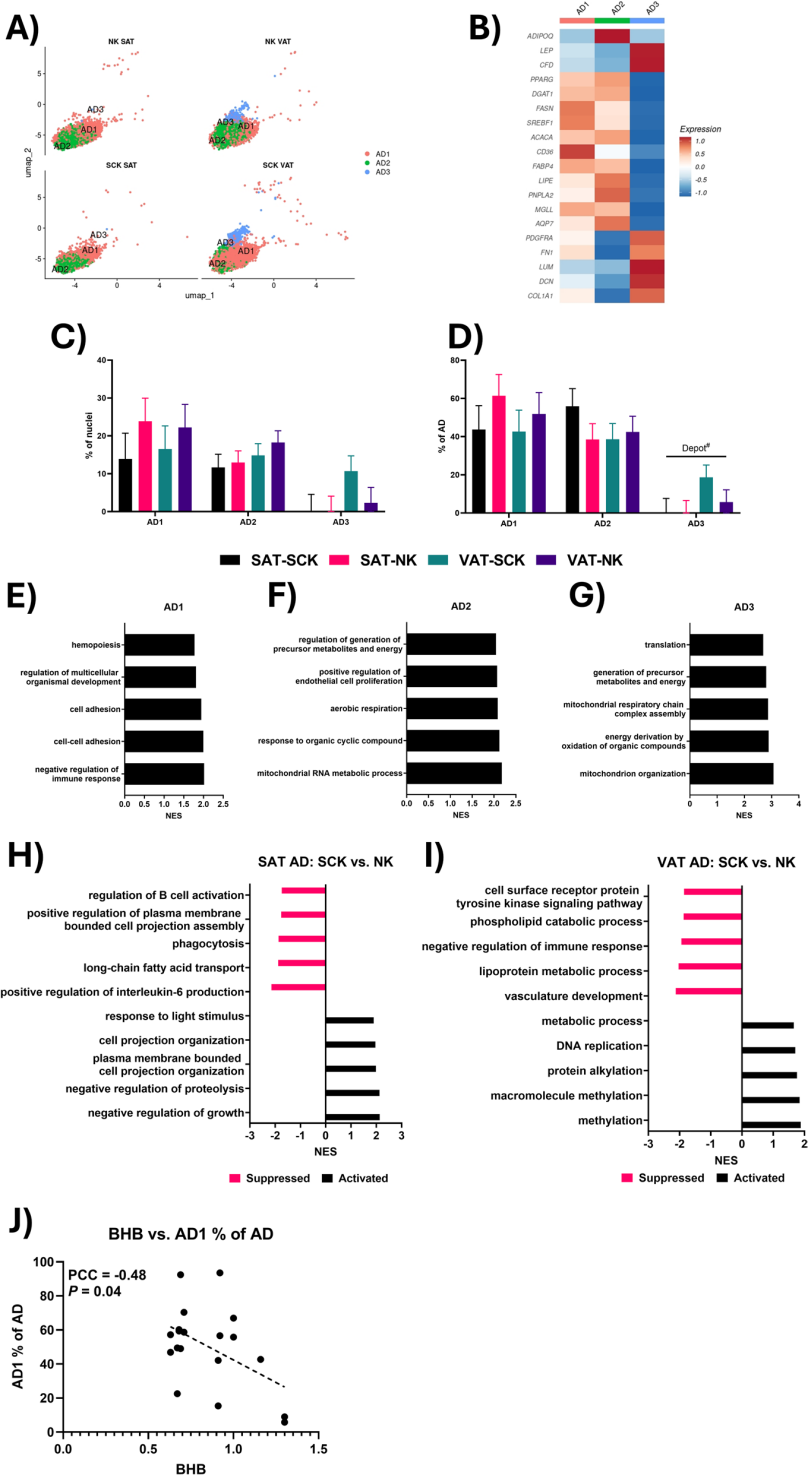
Adipocyte (AD) transcriptional heterogeneity. **A** UMAP of AD clusters in each of the four sample types. **B** Heatmap of typical AD marker gene expression in each of the three identified AD subtypes. **C** Comparison of AD subtype abundance as a % of total nuclei. **D** Comparison of AD subtype abundance as a % of total AD. **E**–**G** Top five Gene ontology (GO) biological process terms, based on net enrichment score (NES), for AD1 (**E**), AD2 (**F**) and AD3 (**G**) in comparison to other AD subtypes. **H** and **I** Top five activated and suppressed GO terms in SAT (**H**) and VAT (**I**) for the comparison between SCK and NK AD. **J** Correlation between serum BHB concentrations and the abundance of AD1 as a % of all AD. Lines over bars indicate the comparison of interest is significant (*P* < 0.05); # indicates a tendency (0.05 ≤ *P* ≤ 0.1). PCC = Pearson correlation coefficient

A key feature of the single-nuclei approach used in our study, as opposed to a single-cell approach, is the ability to sequence and characterize mature adipocyte diversity [12]. As the most abundant cell type in adipose tissue, mature adipocytes store and release energy, secrete adipokines, and interact with immune cells, thereby modulating whole-body metabolic and immune functions [29]. In our database, we identified three distinct clusters of mature adipocytes (Fig. 2A; AD1, AD2, and AD3), which were recognized by the positive expression of *ADIPOQ*, with AD1 as the most abundant, followed by AD2 and AD3. There was no effect of ketosis or depot on the abundances of both AD1 and AD2, which shared a similar gene expression profile, with high expression of adipogenic (*PPARG, FASN, SREBF1*) and lipolytic (*LIPE, PNPLA2, MGLL*) adipocyte signature genes (Fig. 2B). Similar AD subtypes have been identified in previous adipose tissue sn-RNASeq investigations in dairy cattle [11], mice [24, 30] and humans [24, 27]. The top activated GO terms in AD1 compared to all other AD subtypes were highlighted by terms associated with cell adhesion and regulation of the immune response (Fig. 2E), suggesting these cells may be active in adipocyte-immune cell crosstalk. Crosstalk between adipocytes and adipose tissue resident immune cells helps modulate both metabolic and immune functions, with critical roles in inflammation [29]. In contrast, many of the top activated GO terms in AD2 were associated with metabolism and energy utilization, as well as vascular function (Fig. 2F). Adipocytes with high expression of *VEGFA* and *LIPE*, like our AD2 cells (Additional file 1), have been previously described in mice as thermogenic responsive adipocytes [31] and increased *VEGFA* expression by adipocytes has been implicated in “beiging” of white adipose tissue [32]. However, we did not observe any expression of other markers of brown adipose tissue like *UCP1* in the AD2 cluster. Rather, these AD2 cells may represent a subtype of adipocyte involved in adipose tissue remodeling during periods of heightened lipid mobilization, such as during the early postpartum, with increased *VEGFA* and *FGF2* expression supporting angiogenesis [33, 34] as well as proliferation and differentiation of ASPCs [35].

Notably, we identified a depot-specific subtype of adipocytes. AD3, characterized by the high expression of *LEP*, *CFD*, *PDGFRA*, *FN1*, *LUM*, *DCN,* and *COL1A1*, was unique to VAT (6.5% of all nuclei; 12% of all AD), with little to no presence in SAT (0.3% of all nuclei; 0.3% of all AD) (Fig. 2A, C, D). AD3 had lower expression of typical markers of mature AD and lipid metabolism and higher expression of typical ASPC markers and extracellular matrix (ECM) components than the other AD clusters, suggesting this cluster may consist of immature adipocytes early in the differentiation process. A similar cluster of progenitor-like AD were described in the adipose tissue of mice by Sun et al. [36], based on high expression of *CD34* which was also increased in our AD3 cluster (Additional file 1). While enhanced mitochondrial activity, like what was observed among the activated GO terms in AD3 (Fig. 2G), is often used as an indicator of brown or beige fat [37], we did not detect any upregulation of common brown/beige marker genes like *UCP1*, *PPARGC1A*, *EBF2*, *CIDEA* or *DIO2* in the AD3 cluster. However, prior work has demonstrated that as preadipocytes undergo differentiation into mature adipocytes there is a dramatic increase in mitochondrial gene expression and mitochondrial biogenesis in order to support triglyceride synthesis [38].

### SCK promotes progenitor-like profiles in SAT adipocytes, while altering energy metabolism in VAT adipocytes

In SAT, the transcriptional profile of AD from SCK vs*.* NK revealed a suppression of GO terms related to immune responses (Fig. 2H), with decreased expression of *CYBA* as a key driver (Additional file 1). There was also an activation of GO terms associated with cellular projections and growth (Fig. 2H), involving increased expression of *PI16*, *APOD*, and *CD55* (Additional file 1), noted as markers of adipocyte precursor cells [27, 28]. Overall, these findings indicate that in SAT, SCK may trigger a shift in the AD profile to expand and potentially buffer excess circulating lipids generated through increased lipolysis, a process that may also be helping to ameliorate pro-inflammatory conditions.

In VAT, the transcriptome of AD from SCK vs*.* NK revealed a suppression of GO terms associated with vasculature development, lipid metabolism, and negative regulation of immune processes (Fig. 2I), involving increased *VEGFB*, *HLX* and decreased *FGF1* and *APOD* expression (Additional file 1). Prior investigations have demonstrated that *VEGFB* expression can limit white adipose tissue expansion and promote lipolysis, while both *VEGFB* and *HLX* have been implicated in mechanisms associated with the shift from white to brown adipose tissue [39–42]. Furthermore, *APOD*[28] and *FGF1* [43], which were both downregulated, have been implicated as important modulators in maintaining white adipose issue. Together, these results indicate that a ketotic metabolic state promotes changes in energy utilization in VAT AD, while also limiting lipogenesis. Simultaneously, we also observed an activation of GO terms associated with methylation (Fig. 2I), driven by increased expression of *PRMT5* (Additional file 1). As a protein arginine methyltransferase, PRMT5 can modulate both protein function and DNA transcription via methylation [44], with observed roles in promoting healthy lipogenesis in mouse adipocytes [45]. Thus, the upregulation of PRMT5 may reflect an attempt to restore lipogenic function in SCK VAT ADs while other lipogenic pathways and genes are being suppressed.

### Abundance of AD1 negatively correlates with blood BHB concentrations

The primary metric for diagnosing elevated ketone concentrations, BHB is produced as a consequence of increased circulating NEFA that overwhelm the oxidation capacity of the liver [46]. As expected, blood concentrations of BHB were higher in SCK vs*.* NK cows, as described in our recent publication [14]. Interestingly, while all animals had blood NEFA concentrations indicative of increased lipolysis (> 0.5 mmol/L), they did not differ between groups, thus isolating the effect of a ketotic state. We evaluated the associations between distinct metabolic markers in blood and the frequencies of specific cell subtypes to assess how ketosis-associated metabolic changes correlate to specific cell populations. Notably, the abundance of AD1, the most prevalent, classic adipocyte subtype, was negatively correlated with blood BHB, with a lower proportion of AD1 adipocytes in the adipose tissue of dairy cattle with higher BHB concentrations, regardless of adipose tissue depot (Fig. 2J). Given the lipogenic profile of AD1 subtype, their lower abundance may be reflective of an inefficient lipid uptake or simply, a consequence of a lipolytic state within the adipose tissue of dairy cattle in ketotic state.

### Abundance of both adipogenic and fibro-adipogenic ASPCs vary with depot, but not with ketosis

**Fig. 3:**
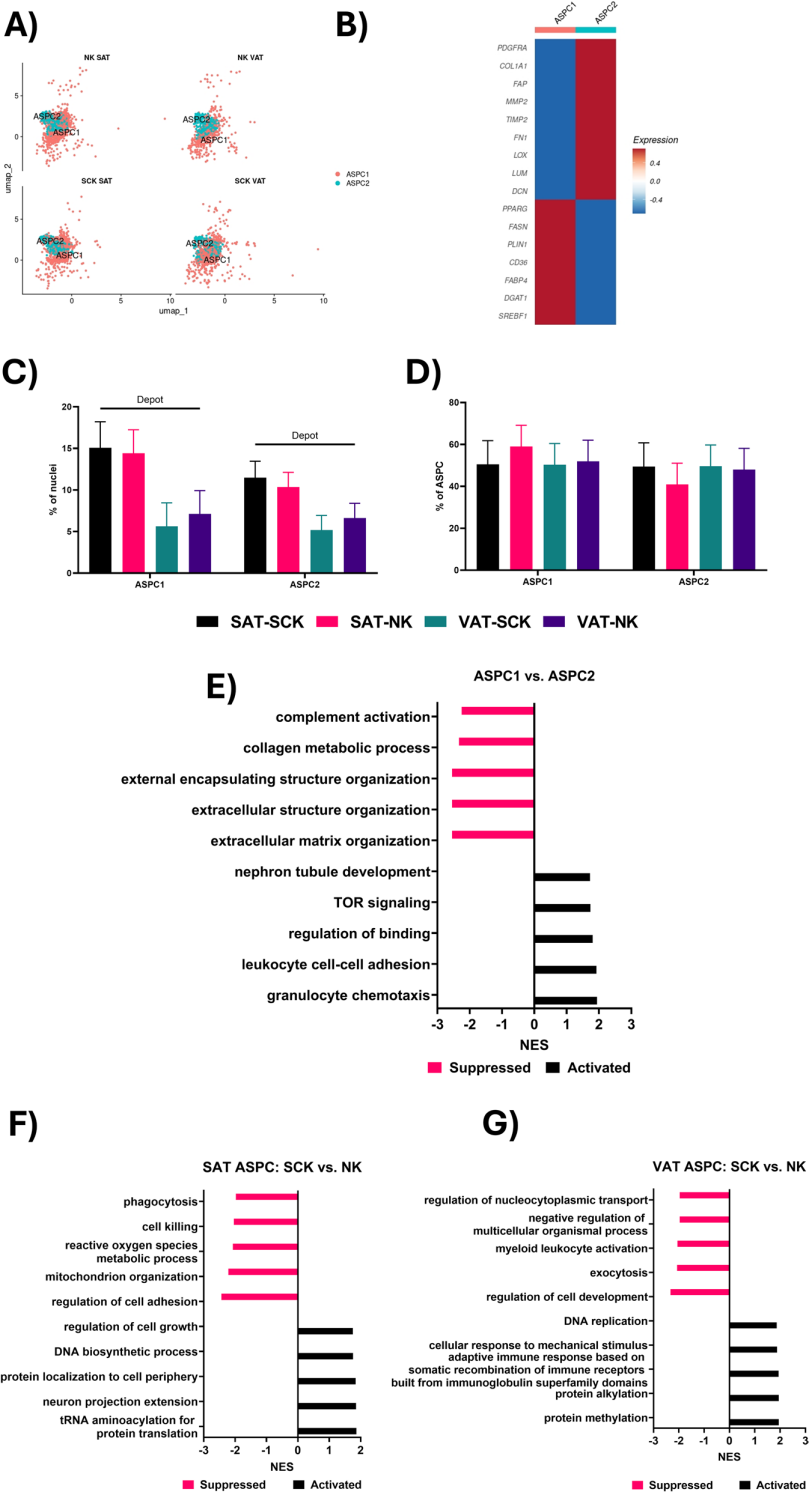
Adipose stem/progenitor cell (ASPC) transcriptional heterogeneity. **A** UMAP of ASPC clusters in each of the four sample types. **B** Heatmap of typical ASPC marker gene expression in each of the two identified ASPC subtypes. **C** Comparison of ASPC subtype abundance as a % of total nuclei. **D** Comparison of ASPC subtype abundance as a % of total ASPC. **E** Top five activated and suppressed Gene ontology (GO) biological process terms, based on net enrichment score (NES), for the comparison between ASPC1 and ASPC2. **F** and **G** Top five activated and suppressed GO terms in SAT (**F**) and VAT (**G**) for the comparison between SCK and NK ASPC. Lines over bars indicate the comparison of interest is significant (*P* < 0.05); # indicates a tendency (0.05 ≤ *P* ≤ 0.1)

The ASPC within adipose tissue have been the focus of continued research efforts due to their ability to proliferate and differentiate into mature AD [47], facilitating adipose tissue growth and expansion, as well as their contributions to tissue remodeling [48, 49]. Importantly, the proliferation and differentiation of these ASPC into AD, as opposed to hypertrophic expansion of pre-existing AD, has been associated with healthy adipose tissue expansion [50]. The two ASPC clusters identified (Fig. 3A) in our study represent two distinct progenitor cell subtypes, as previously reported in cattle [11], mice [24, 51] and human adipose tissue [24, 28]. ASPC1 have an adipogenic gene profile with high expression of *PPARG*, *FASN*, *PLIN1*, *CD36*, *DGAT1*, and *SREBF1,* compatible with committed preadipocytes [52]. In contrast, ASPC2 has a fibrogenic profile marked by high expression of *PDGFRA*, *COL1A1*, *FAP*, *MMP2*, *TIMP2*, *FN1*, *LOX*, *LUM*, and *DCN* (Fig. 3B), compatible with fibro-adipogenic progenitors (FAP)[28, 52]. Interestingly, both ASPC subtypes were more abundant in SAT compared to VAT (Fig. 3C), with no effect of SCK within each depot or across all samples. However, no differences were observed in the overall ASPC composition, with each ASPC type making up ~ 50% of all ASPCs in each group (Fig. 3D).

Both ASPC populations identified in our study share similarities with other ASPCs described in previous investigations. The more committed/adipogenic ASPC1 population closely reflects the ASPC1 population described by Michelotti et al. [11] in adipose tissue of dairy cattle, exhibiting higher expression of *PPARG* and other adipogenic genes. The more uncommitted/fibrogenic ASPC2s in our study also align with the ASPC2 and ASPC3 clusters identified by Michelotti et al. [11] in VAT and SAT of dairy cows, characterized by higher expression of *PDGFRA*, *COL1A1*, and other ECM genes. Distinct ASPC subtypes in VAT of mice were also described by Hepler et al. [51], including fibro-inflammatory progenitors (FIPs) similar to the ASPC2s in our study, characterized by high expression of fibrosis and inflammation-related genes, and committed preadipocytes similar to the ASPC1s in our study characterized by high expression of lipogenic genes. Thus, the increased abundance of both ASPC subtypes in SAT compared to VAT in our study suggests a greater capacity for adipogenesis, adipose tissue expansion, and remodeling in SAT.

The functional analysis of the comparison between ASPC1 and ASPC2 supported their gene profiles, with a suppression of ECM and collagen related GO terms in ASPC1 compared to ASPC2 (Fig. 3E). However, while the expression of genes associated with the complement system (*C4A*, *CFB*, *C7*) was higher in ASPC2 (Additional file 2), genes related to immune cell chemotaxis and adhesion (*S100A9*, *ITGB2*, *GATA3*) were upregulated in ASPC1 (Additional file 2), suggesting both cell types may be performing immunomodulatory functions.

Our functional analysis of the SCK and NK ASPCs in SAT identified a suppression of GO terms related to immune function and cell adhesion (Fig. 3F), associated with reduced expression *TGFB1* and *LEP* (Additional file 2). Concomitantly, there was an activation of GO terms associated with cell growth and protein metabolism (Fig. 3F), with increased *CDK5* expression implicated in all the activated terms (Additional file 2). Both *TGFB1* and *CDK5* have been studied for their roles in regulating ASPC differentiation and adipogenesis. While *TGFB1* inhibits ASPC commitment to adipocytes [52], *CDK5* has been associated with dysregulated PPARγ signaling in obesity [53]. The dysregulation of PPARγ by *CDK5* is also implicated in adipose tissue insulin resistance, with CDK5-mediated phosphorylation of PPARγ, a master regulator of adipogenesis, suppressing *ADIPOQ* expression [53] as observed in our data (Additional file 2). The decreased expression of *LEP*, associated with suppressed immune GO terms, is also of note, as leptin is known to have a general pro-inflammatory effect on immune cells [54, 55]. The expression of *LEP* is also associated with adipogenic differentiation [56]. Together, our data suggest an impaired adipogenic profile of SAT SCK ASPCs that may also be associated with a dysregulated adipose tissue immune environment.

In contrast to what we observed in the SAT, in VAT ASPCs, *CDK5* was among the top downregulated genes in SCK vs. NK (Additional file 2) and was associated with the majority of the top suppressed GO terms (Fig. 3G). Similar to the functional analysis results in the VAT SCK ADs, GO terms associated with methylation and alkylation were activated in VAT ASPCs from SCK cows (Fig. 3G), driven by increased expression of *PRMT5* (Additional file 2), indicating an improved adipogenic capacity. Together, our results suggest that SAT and VAT ASPCs transcriptomes are differentially affected by a ketotic state, with a potentially impaired adipogenic response in SAT and an enhanced adipogenic response in VAT. While adipose tissue expansion via hyperplasia is preferred over hypertrophic expansion [50], increased visceral adiposity is associated with increased inflammation and poor health outcomes in humans [57]. Thus an increase in visceral adipose tissue adipogenic capacity may be linked to the increased incidence of other diseases often associated with subclinical ketosis in dairy cattle [5].

### Macrophages comprise most immune cells in adipose tissue

**Fig. 4:**
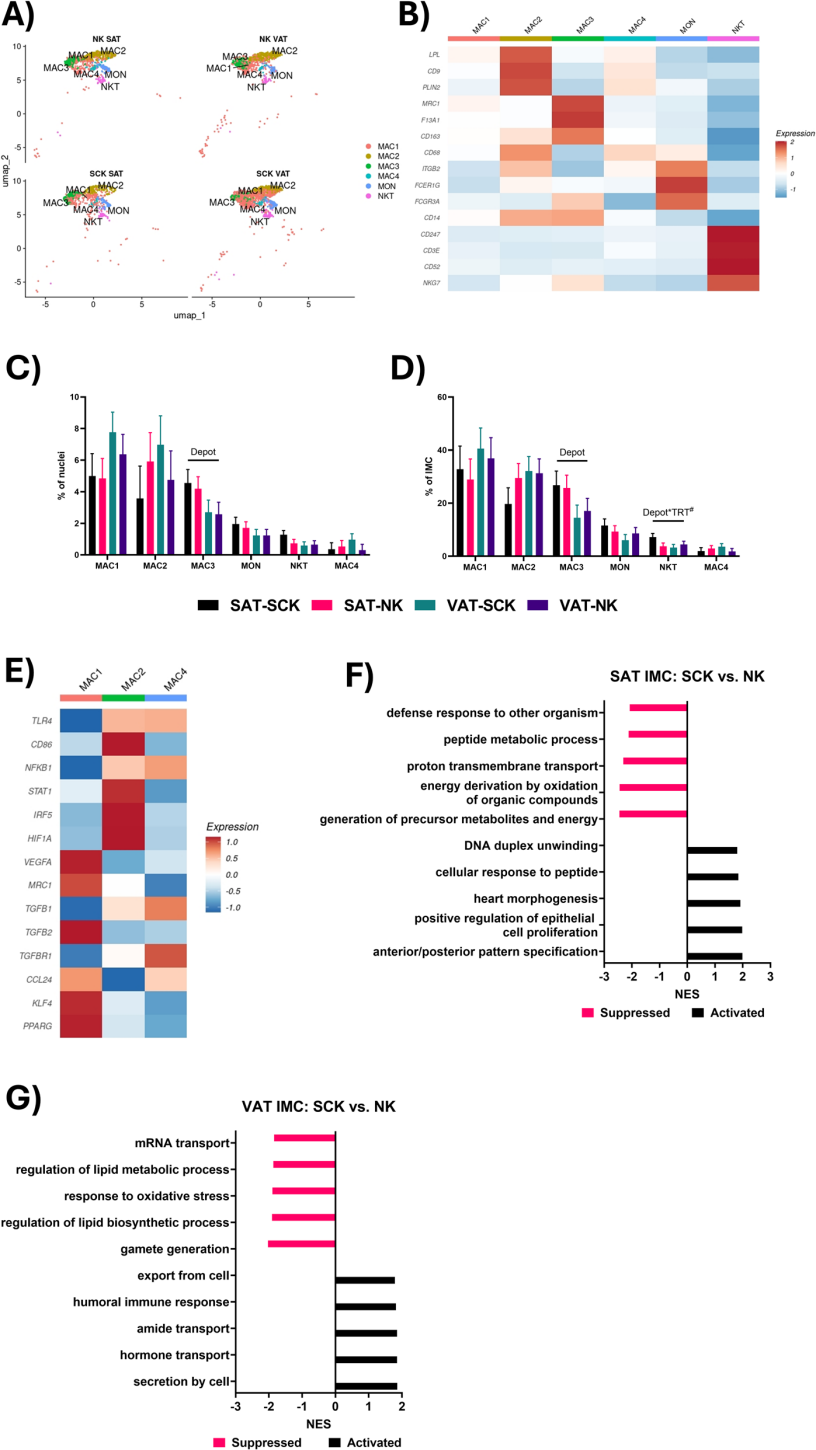
Immune cell (IMC) transcriptional heterogeneity. **A** UMAP of immune cell clusters in each of the four sample types. **B** Heatmap of typical immune cell marker gene expression in each of the six identified immune cell subtypes. **C** Comparison of immune cell subtype abundance as a % of total nuclei. **D** Comparison of immune cell subtype abundance as a % of total IMC. **E** Heatmap of pro- and anti-inflammatory marker gene expression in each of the three lipid-associated macrophage (LAM) clusters. **F** and **G** Top five activated and suppressed GO terms in SAT (**F**) and VAT (**G**) for the comparison between SCK and NK IMC. Lines over bars indicate the comparison of interest is significant (*P* < 0.05); # indicates a tendency (0.05 ≤ *P* ≤ 0.1)

Immune cells play critical roles in the function of adipose tissue by modulating inflammation, lipid metabolism, and insulin sensitivity as well as in the remodeling of the adipose tissue ECM and vasculature [58–60]. Our data show a great diversity of immune cell types in adipose tissue of periparturient dairy cows. Among the six immune cell clusters identified in the sn-RNASeq dataset (Fig. 4A), we identified four types of macrophages, with MAC1, MAC2 and MAC4 characterized as lipid-associated macrophages (LAM; *LPL*, *CD9*), and MAC3 as perivascular macrophages (PVM; *MRC1*, *F13A1*), one cluster of monocytes (MON; *FCER1G*, *FCGR3A*), and one cluster of mixed natural killer/T cells (NKT; *CD247*, *CD3E*, *CD52*, *NKG7*) (Fig. 4B).

In agreement with prior studies in dairy cattle [11], mice [24, 30] and humans [24, 26, 27], macrophages made up the majority of immune cells in our SAT and VAT. Prior studies show increases in the percentage of macrophages in adipose tissue of dairy cattle with clinical ketosis [61], and in early postpartum associated with increased loss of body condition score [62]. Interestingly, we saw no effects of SCK on the abundance of macrophages in either depot (Fig. 4C and D). This contrast may suggest a mild effect of SCK on local adipose tissue immune cell infiltration in our study compared to more chronic states of metabolic dysfunction, such as clinical ketosis and postpartum body condition score loss reported in prior studies, which effects may be more evident. Rather, we observed transcriptome changes in MACs from SCK and NK, which suggest that a mild ketotic state affects function rather than quantity of immune cells in adipose tissue, with depot-specific differences.

Notably, independent of ketotic state, we identified abundant LAM in the adipose tissue of periparturient cows, which shared similar gene expression profiles with the MAC4 cluster described by Michelotti et al. [11] in dairy cattle. Lipid-associated macrophages are specialized macrophages characterized by their close association with adipocytes. Through their interactions with adipocytes, these LAMs can modulate adipocyte metabolism, insulin sensitivity and inflammation [63]. Notably, the three LAM clusters identified in our investigation appear to represent three different phenotypes (Fig. 4E). The MAC1 LAM have lower expression of pro-inflammatory markers like *TLR4*, *CD86* and *NFKB1*, and higher expression of anti-inflammatory markers including *VEGFA*, *MRC1*, and *PPARG,* resembling alternatively activated macrophages associated with tissue repair [64]. In contrast, MAC2 LAM cluster had the opposite transcriptional profile, with increased expression of pro-inflammatory markers and decreased expression of anti-inflammatory markers (Fig. 4E). The third population of lipid-associated macrophages (MAC4) were the least abundant immune cell identified in the adipose tissue samples in our study and appear to represent a more heterogenous population of lipid-associated macrophages, or a population undergoing phenotypic changes, as indicated by mixed expression of pro-inflammatory (*TLR4*, *NFKB1*) and anti-inflammatory (*TGFB1*, *TGFBR1*, *CCL24*) marker genes.

Also similar between our study and the data described by Michelotti et al. [11], were their MAC2 cluster and the MAC3 cluster in our own dataset, characterized as perivascular macrophages (PVM). A similar group of PVMs were described by Hildreth et al. [26] in human adipose tissue, characterized by high expression of *LYVE1*, *SELENOP*, and *C1QA*, with both *SELENOP* and *C1QA* also highly expressed in the PVMs identified in our study. However, while Michelotti et al. [11], found these PVM in similar abundances between SAT and VAT in their dairy cow adipose tissue samples, there were significantly more in SAT compared to VAT in our investigation. However, while the samples used by Michelotti et al. [11] were collected at an abattoir from dairy cows of unknown backgrounds, our samples were collected from live dairy cattle during the early postpartum period. Given that PVMs are implicated in remodeling of the adipose tissue vasculature [65], the increased abundance of these cells in SAT compared to VAT provides additional evidence of an increased capacity for remodeling in this adipose tissue depot.

The MON cluster in our data closely resembles the non-classical monocytes (ncMON) described by Hildreth et al. [26] and Massier et al. [27] in their human datasets, characterized by low expression of *CD14* and high expression of *FCGR3A* and *TCF7L2*. Prior investigations in humans have described positive associations between this subtype of monocytes and obesity, with adipose tissue from obese patients containing higher amounts of nonclassical monocytes than in adipose tissue from lean patients [66, 67]. Notably, Poitou et al. [67] also observed a positive correlation between the abundance of these nonclassical monocytes and insulin resistance (HOMA-IR). The fact that insulin resistance is a common phenomenon experienced by early lactation dairy cattle [68], may partly explain why we observed a higher abundance of this monocyte subtype in our data than what has previously been described in dairy cattle [11], mice [24, 30], and humans [24, 26].

The natural killer and T cells (NKT) identified in our study share similarities to the NKT cluster identified by Michelotti et al. [11] in their SAT and VAT from dairy cattle. The presence of NK and T cells have also been described in adipose tissue in humans [24, 26, 27] and mice [24, 30], although some markers used to further define subtypes of T cells and NK cells were not identified in our dataset. Notably, we observed a tendency for a Depot × Treatment interaction in the abundance of the NKT cluster as a % of all IMC, with SCK tending to increase the abundance of NKT in SAT and decrease the abundance of NKT in VAT (Fig. 4D).

### The ketotic state is associated with anti-inflammatory changes in SAT IMCs and pro-inflammatory responses in VAT IMCs

Comparing the transcriptomes of IMCs in SCK SAT to NK SAT, we observed suppression of GO terms associated with metabolic activity (Fig. 4F), driven by decreased expression of genes associated with mitochondrial respiration (Additional file 3). As reviewed by Angajala et al. [69], mitochondrial metabolism plays an important role in immune cell function, increasing glucose utilization to execute inflammatory functions. Concomitantly, we observed the activation of GO terms related to cellular development and proliferation (Fig. 4F), mediated by increased expression of *BMP4* and *TGFBR1* (Additional file 3) [69], supporting the notion of an anti-inflammatory response in SAT of cows in a ketotic state, performed by pro-resolving macrophages [70–72] and T cells[73, 74], perhaps as a homeostatic mechanism to maintain adipose tissue function.

Among the top suppressed GO terms in VAT IMCs from cows in a ketotic state were those associated with lipid metabolism and oxidative stress (Fig. 4G), including decreased gene expression of *LDLR*, *PCK1*, and *EGR1* (Additional file 3). Notably, activated GO terms were associated with secretion and transport (Fig. 4G), showing increased expression of *AQP1*, *PFKL*, and *CLTRN* (Additional file 3). Increased expression of both, *EGR1* and *PCK1,* have been associated with anti-inflammatory profiles in macrophages [75, 76], while *LDLR*, is implicated in cholesterol uptake and foam cell formation [77]. Furthermore, *AQP1* and *PFKL* have both been associated with pro-inflammatory responses in immune cells [78, 79]. Overall, these results indicate that SCK induces a more pro-inflammatory immune cell profile in VAT, opposite of what we observed in SAT.

### Endothelial cells are diverse cell types in both SAT and VAT

**Fig. 5:**
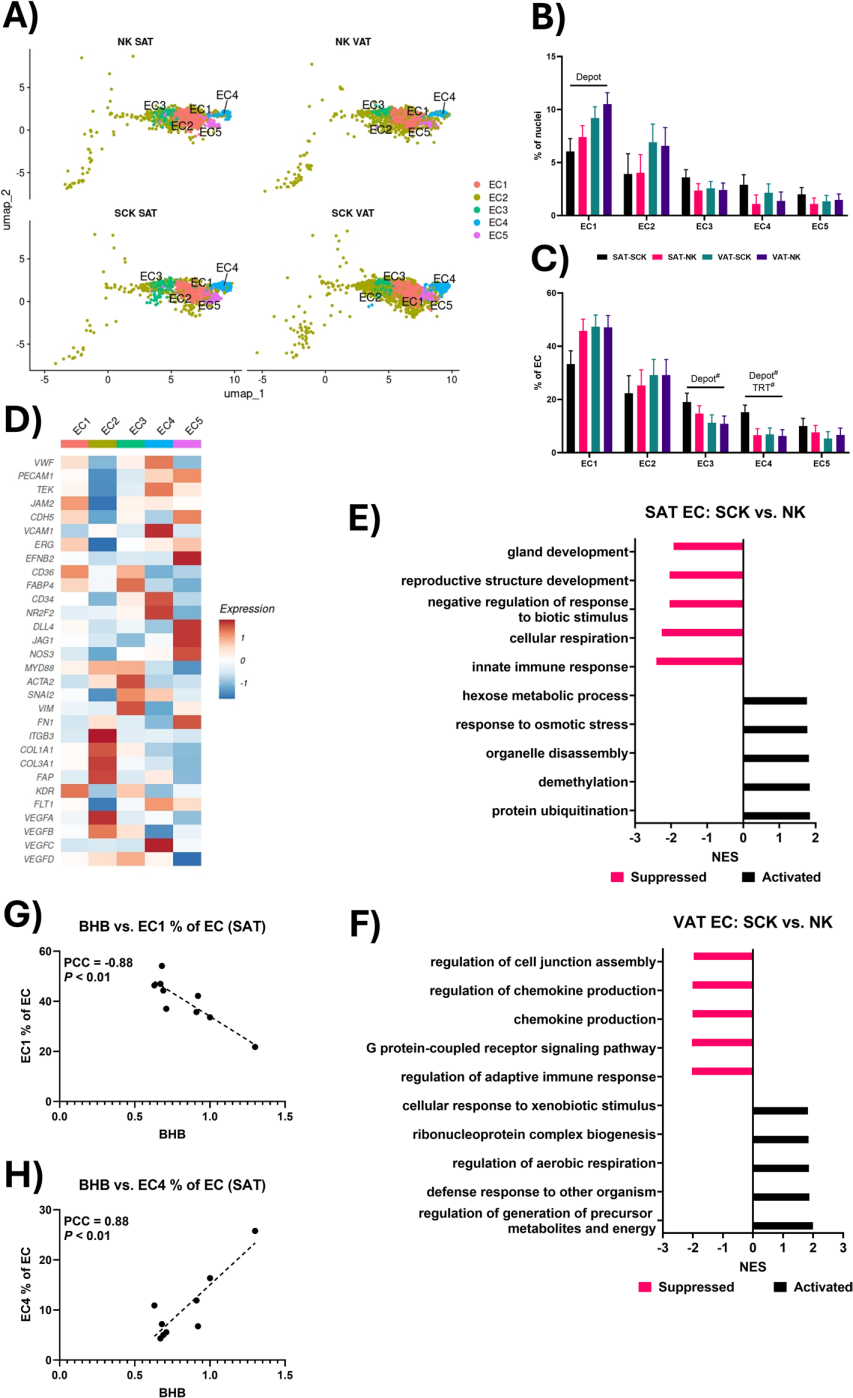
Endothelial cell (EC) transcriptional heterogeneity. **A** UMAP of endothelial cell clusters in each of the four sample types. **B** Comparison of endothelial cell subtype abundance as a % of total nuclei. **C** Comparison of endothelial cell subtype abundance as a % of total EC. **D** Heatmap of typical EC marker gene expression in each of the five EC clusters. **E** and **F** Top five activated and suppressed GO terms in SAT (**E**) and VAT (**F**) for the comparison between SCK and NK EC. **G** Correlation between serum BHB concentrations and the abundance of EC1 as a % of all EC. **H** Correlation between serum BHB concentrations and the abundance of EC4 as a % of all EC. Lines over bars indicate the comparison of interest is significant (*P* < 0.05); # indicates a tendency (0.05 ≤ *P* ≤ 0.1). PCC = Pearson correlation coefficient

Endothelial cells (EC), which comprise the vascular network within adipose tissue, play a critical role in modulating the metabolic, inflammatory, and remodeling processes within adipose tissue [80]. Among the five EC clusters identified in our investigation (Fig. 5A), EC1 was the only cluster significantly affected by depot, being more abundant in VAT than SAT (% of total nuclei; Fig. 5B). Endothelial cells in EC1 cluster had high expression of vascular endothelial cells markers like *VWF*, *PECAM1*, *TEK* and *JAM2,* as well as markers of microvascular ECs, *CD36* and *FABP4* (Fig. 5D). Similar EC types have been identified in the adipose tissue of dairy cattle[11] and humans [27, 28, 81]. As shown by Gogg et al. [82], microvascular EC crosstalk with preadipocytes to support adipogenic differentiation and express high *CD36* and *FABP4* after extended exposure to increased fatty acid concentrations, such as during heightened lipolysis [82]. As such, compared to SAT, the increased abundance of EC1 in VAT may indicate an elevated lipolytic activity [83, 84], potentially increasing the capacity of microvascular EC to handle fatty acids. In contrast, Michelotti et al. [11] reported greater abundance of microvascular EC in SAT compared to VAT samples of dairy cows. As discussed for many other discrepancies between our findings and theirs, this may be attributable to the metabolic status of the cattle from which the samples were collected. While adipose tissue samples utilized by Michelotti et al. [11] were from cattle processed at an abattoir and animal history was not available, the samples collected in our study were from verified early lactation cattle, where enhanced lipolysis is typical [3].

The EC2 cluster presents a unique transcriptional profile with low expression of typical EC markers (ex. *VWF*, *PECAM1*, *TEK;* Fig. 5D) and higher expression of genes associated with the endothelial-mesenchymal transition [85, 86] (EndoMT), including *ACTA2*, *FN1*, *ITGB3*, *COL1A1*, *COL3A1*, and *FAP*. A similar subset of EndoMT were identified in porcine adipose tissue [87], although the role these transitional cells play in modulating adipose tissue function requires further investigation [88].

The cells within the EC3 cluster also likely represent EndoMT cells, perhaps at a different point of differentiation than EC2, with a tendency for a greater proportion (% of total EC) in SAT compared to VAT (Fig. 5C). While EC3 expressed high *ACTA2*, *SNAI2*, and *VIM*, markers of EndoMT cells (Fig. 5D), they also have increased expression of *CD36* and *FABP4*, indicating a potential role in microvascular function. Together, the increased expression of the transcription factor *SNAI2*, implicated in stimulating EndoMT [89], as well as the maintained expression of microvascular EC markers indicate that EC3 may represent an earlier stage of the EC2 EndoMT.

The endothelial cell cluster EC4 was characterized by high expression of typical vascular EC markers like *VWF*, *TEK*, and *VCAM1.* Notably, EC4 had lower expression of *CD36* and *FABP4* than the EC1 cells and with higher expression of *CD34* and *NR2F2* (Fig. 5D), which are markers of endothelial progenitor cells [90, 91]. As a % of total EC, the EC4 cluster tended to be more abundant in SAT than VAT, and in SCK vs*.* NK. As such, the EC4 cells most likely represent a subpopulation of differentiating vascular EC.

The least abundant EC subtype was the EC5, characterized by higher expression of mature EC markers (*PECAM1*, *CDH5;* Fig. 5D). Endothelial cells in EC5 cluster also expressed high *DLL4* and *JAG1,* implicated in the spatial regulation of endothelial sprouting [92, 93] and vessel maturation [94], as well as *EFNB2* [95] and *NOS3* [96], associated with increased angiogenesis. These results indicate EC5 cells are likely proliferative sprouting/angiogenic EC.

Notably, absent in our investigation were lymphatic endothelial cells (LEC), typically characterized by increased expression of *LYVE1*, *MMRN1*, and *PROX1*. In their dairy cow study, Michelotti et al. [11] found that these cells only comprised 0.3% of all adipose tissue cells, with other human studies also finding them in low abundance [28, 30].

### Ketotic state affects genes associated with inflammation and angiogenesis in both SAT and VAT endothelial cells

Functional transcriptional analysis of SAT EC of SCK vs*.* NK cows identified suppressed GO terms associated with the immune response and cellular development, including the decreased expression of *CEBPB* and *SERPINF1*, implicated in vascular inflammation [97, 98] and angiogenesis inhibition [99, 100], respectively (Additional file 4). Notably, many genes associated with the complement system and innate immune response, including *C1QA* and *C1QB*, as well as *IL34* were also downregulated in SAT EC of SCK vs*.* NK (Additional file 4), suggesting a reduced inflammatory profile in a ketotic state. Together, these data suggest SCK promotes an anti-inflammatory EC profile in SAT, while also possibly promoting angiogenesis.

Within VAT, suppressed GO terms in EC from SCK vs*.* NK cows were related to chemokine signaling and cell junctions (Fig. 5F), driven by decreased expression of *EGR1* and *SNAI2* (Additional file 4). Activated GO terms were associated with mitochondrial metabolism and the defense response (Fig. 5F), driven by increased expression of *TNFAIP8L2*, *CFB* and *SHMT2* (Additional file 4). Interestingly, *EGR1*, an upstream mediator of *VEGF* expression [101] in ECs, was also implicated in the suppressed pathways of the SCK VAT IMCs, and *SNAI2* is involved in the EndoMT process [89], indicating that SCK may be suppressing angiogenesis and endothelial cell maturation in VAT. In agreement with this notion, is the high expression of *TNFAIP8L2* (encoding TIPE2), which was the top upregulated gene (> 6.6 log_2_FC) in VAT EC from SCK vs*.* NK cows (Additional file 4). The activity of TIPE2 has been associated with decreased expression of VEGF [102, 103], as well as in suppression of the immune response [104], likely playing a role in suppressing the chemokine and adaptive immune system-related GO terms we observed in our analysis (Fig. 5F; Additional file 4). Overall, a ketotic state appears to enhance an anti-angiogenic effect on VAT EC, with indications of a reduced inflammatory profile as well.

### Circulating ketones are associated with the abundance of specific EC clusters in SAT

Our analysis revealed a negative association between serum BHB concentrations and the abundance of EC1 (Fig. 5G) and positive correlation with the abundance of EC4 (Fig. 5H) in SAT, but not VAT. This may indicate a shift in the composition of EC in SAT driven by a systemic ketotic state. Namely a shift from more mature, lipid-handling microvascular EC1 towards more progenitor-like EC4. While studies evaluating the direct effects of BHB on endothelial cell function are limited, Wu et al. [105] reported a protective effect of BHB treatment on aortic endothelial cell injury in diabetic rats, with increased VEGF suggested as a potential mechanism. As such, the heightened circulating BHB concentrations in dairy cows in a ketotic state in our investigation may be promoting the generation of new endothelial cells in SAT as a potential homeostatic mechanism to restore adipose tissue function in a pro-lipolytic state.

### Cell–cell communication through collagen, laminin and VEGF signaling is affected by ketosis

We used CellChat to analyze overexpressed genes and predict ligand-receptor (L-R) interactions within NK SAT, NK VAT, SCK SAT and SCK VAT. Although the gene symbols for the human and bovine genomes are largely similar, some genes and pathways with species-specific differences in annotation (i.e. the major histocompatibility complex genes in humans (*HLA*) and bovine (*BoLA*)) cannot be assessed using the human CellChat database.

**Fig. 6:**
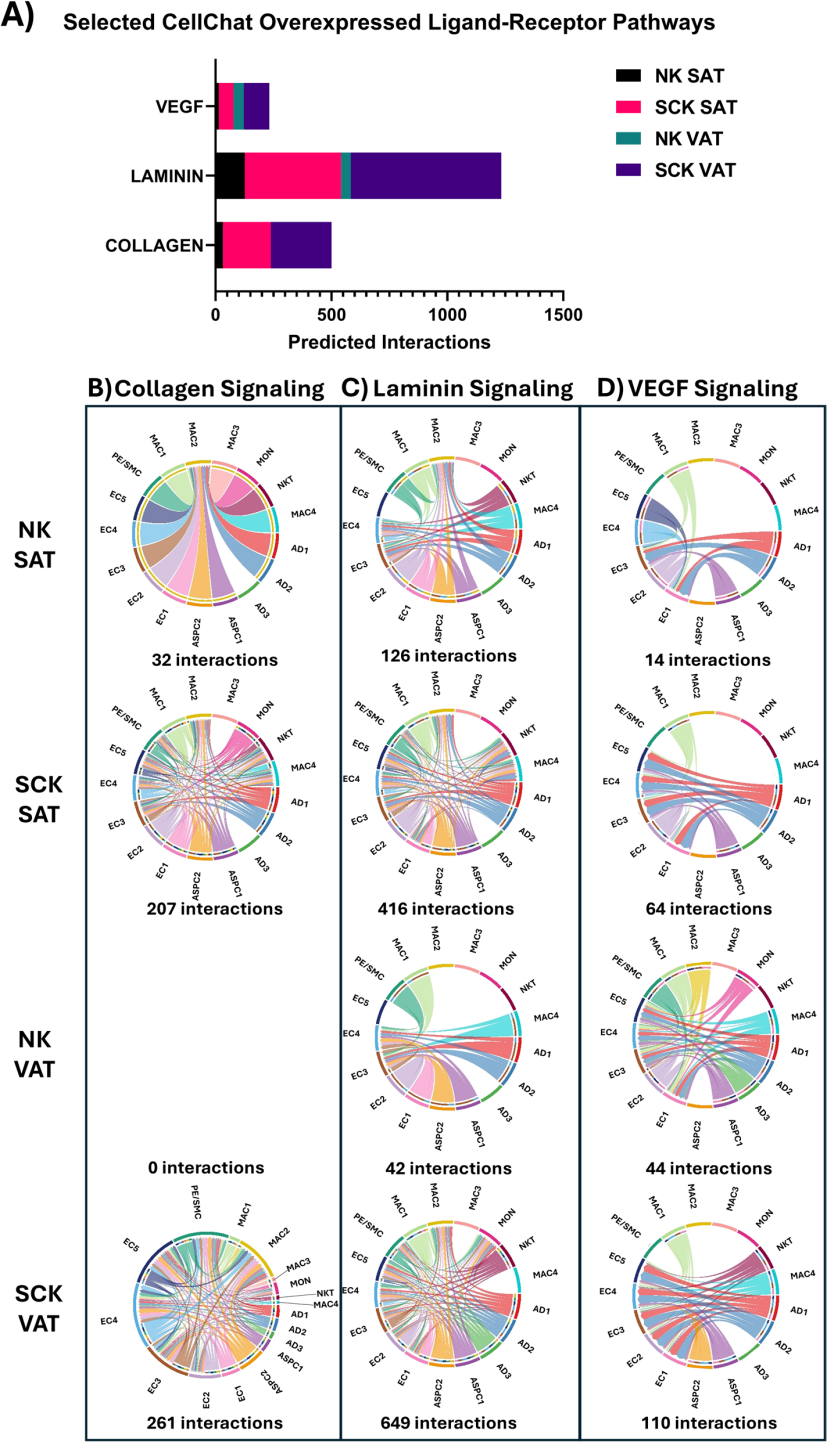
CellChat results for predicted ligand-receptor (L-R) interactions in NK SAT, SCK SAT, NK VAT, and SCK VAT. **A** Total number of predicted interactions in each of the four sample types for VEGF, laminin and collagen signaling pathways. **B**–**D** Chord diagrams of predicted interactions and total number of interactions between cell types in each of the four sample types for (**B**) Collagen signaling, (**C**) Laminin signaling, and (**D**) VEGF signaling. Arrows between clusters indicate predicted signaling from a ligand to a receptor

The VEGF, laminin, and collagen signaling pathway networks had the highest number of ligand-receptor interactions across all groups (Fig. 6A). Overall, the ketotic state enhanced the number of predicted interactions in both depots compared to non-ketotic cows for all three highlighted pathways (Fig. 6A, Additional file 5). The identification of VEGF, laminin and collagen signaling as the most prevalent L-R interactions in our dataset is likely a reflection of the dynamic remodeling changes in adipose tissue required for early postpartum dairy cattle to adapt to the demands of lactation [2, 3]. As reviewed by Contreras et al. [2], the dramatic increase in lipolysis experienced by dairy cattle during the early lactation period is central in remodeling of the adipose tissue, including changes in immune cell infiltration, cellular turnover, and extracellular matrix (ECM) structure.

As the primary component of the adipose tissue ECM, collagens are important structural and signaling molecules that participate in a wide range of adipose tissue functions [106]. We have recently demonstrated increased collagen concentrations and turnover in SAT vs*.* VAT of dairy cows, accompanied by decreased tissue stiffness and higher adipogenic capacity [13]. Indeed, collagen signaling through integrins is associated with the differentiation of preadipocytes into mature adipocytes [107, 108], modulating angiogenesis [108, 109], and regulating immune cell migration and function [110]. Interestingly, VAT of cows with SCK had the greatest number of L-R interactions within the collagen signaling pathway (Fig. 6B, Additional file 5), while no L-R interactions were predicted in VAT of NK cows. This finding indicates a more pronounced effect of ketosis on collagen signaling in VAT vs*.* SAT.

Like collagens, laminins play key structural and signaling roles in the ECM [111]. Laminin signaling via integrins are associated with adipogenic differentiation [112], cell migration [113, 114], and angiogenesis [108]. Our results demonstrate that, across all groups, the majority of laminin signaling interactions were predicted between integrins expressed by EC and laminins expressed by most other cell clusters, with *CD44* functioning as a laminin receptor in the immune cells clusters (Fig. 6C, Additional file 5). Notable was the apparent depot-specific effect of ketosis. While VAT had a greater number of predicted interactions compared to SAT in cows with SCK, in non-ketotic cows, SAT had a greater number of predicted interactions than VAT, suggesting that the effects of ketosis on laminin signaling may be more pronounced in VAT than in SAT.

Proper angiogenesis, primarily facilitated by VEGF signaling through VEGF receptors, is critical for adipose tissue homeostasis. As the primary route of nutrients into and out of adipose tissue, the vascular network plays important roles in adipogenic differentiation, inflammation, and hypoxia [80, 115–118]. The potential impact of SCK on vascular changes within adipose tissue was highlighted by an increase in the number of predicted L-R interactions in the VEGF signaling pathway network in both SAT SCK and VAT SCK relative to cows without ketosis (Fig. 6D, Additional file 5). Together, these data further support the association between SCK and heightened adipose tissue remodeling, with a particular emphasis on the vascular network.

## Discussion

The findings presented herein provide novel insight into the cellular heterogeneity of SAT and VAT of early lactation dairy cattle, as well as into how SCK affects the transcriptional heterogeneity of adipose tissue cells at the single nuclei level. While prior studies utilizing sn-RNASeq to assess the cellular heterogeneity of adipose tissue depots in dairy cattle are limited, the cell types identified in this investigation are consistent with what was identified by Michelotti et al. [11] in the dairy cow SAT and VAT. Furthermore, the abundance of the different types of cells identified within the SAT and VAT of the dairy cattle used in this study align with what has previously been described in dairy cattle [11], humans [24, 27], and mice [24], with ADs and ASPCs making up the majority of adipose tissue cells, followed by EC, IMC and PE/SMC. However, while other studies have identified mesothelial cells as a VAT-specific cell type, mesothelial cells were not detected in the adipose tissue samples used in our study. This contrast between our study and previous investigations, particularly our previous sn-RNASeq study in dairy cows [11], may be due to the inconsistency of anatomical location of sample collection using different techniques, given that the protocols for sample handling and tissue digestion, as well as the sequencing, quality control, and analysis methods were nearly identical between studies. Sample collection via laparotomy in live animals limits the ability to precisely collect anatomically identical samples from each animal, perhaps excluding the mesothelial layer during sample collection. On the other hand, postmortem collection allows for full visualization of fat depots, providing greater consistency among samples, including collection of the mesothelial layer.

Although SCK did not affect the abundance of any of the identified cell types in our adipose tissue samples, we identified underlying transcriptional changes associated with the response to subclinical ketosis in each adipose tissue depot. The observed changes in gene expression profile of depot-specific cell type in SCK vs. NK cows, rather than changes in the abundance of cell types, may reflect subtle effects of a ketotic metabolic state. Within this context, increases in adipose tissue lipolysis (reflected in high circulating NEFA) and circulating concentrations of BHB may alter the gene expression profile of adipose tissue cells, as an adaptive mechanism, without dramatically altering cellular abundance. In this study, we did not assess ketonemia dynamics, thus, we were unable to define whether the detected increased BHB concentrations were persistent, and whether they reflect the beginning or the end of a clinical ketosis case. Notably, in vitro treatment with both NEFA [119] and BHB [120–122] alters adipose tissue gene expression in a depot-specific manner. Similarly, the depot-specific transcriptomic effects of subclinical ketosis in subcutaneous and visceral adipose tissue were highlighted in our analysis. Interestingly, protein expression of HCAR2, the receptor for BHB was found to be higher in subcutaneous compared to visceral adipose tissue explants from dairy cattle [123] and likely also contributes to depot-specific responses to ketotic conditions.

Within SAT, our findings indicate that a ketotic state is associated with transcriptional changes encoding reduced inflammatory profile, enhanced adipogenesis, and increased angiogenesis compared to non-ketotic cows. Despite being contradictory to our expectations, influenced by previous literature demonstrating a greater degree of systemic inflammation in cows experiencing SCK [124, 125], this may reflect a homeostatic mechanism to reduce local adipose tissue dysfunction. Contributing to this notion of recovery of homeostasis were the increases in adipogenesis-associated genes and terms in the AD and ASPC clusters in SAT of cows with ketosis. Given the enhanced lipolysis experienced by early lactation dairy cattle, improving adipogenesis has been proposed as a potential mechanism to buffer the increase in circulating lipids [2]. Interestingly, Ford et al. [126], showed that PBMC-conditioned media from cows with SCK stimulated bovine preadipocyte proliferation and increased lipogenic gene expression, providing further evidence of an stimulatory effect of SCK on adipogenesis. Similar effects may be underlying our results, where improved adipogenesis in SCK functions to buffer excess mobilized lipids and may partially explain the lack of differences in circulating NEFA concentrations between SCK and NK cows. Supporting these apparent homeostatic mechanisms in SCK SAT may be the enhanced remodeling activity proposed in the CellChat results. The tremendous increase in the number of predicted L-R interactions in SAT of SCK compared to NK indicates a greater degree of communication between ECM and cells, and through VEGF, providing key signals for adapting to changes in adipose tissue structure and function.

In contrast to SAT, where homeostatic mechanisms induced by SCK may be working to restore tissue function, the effects of SCK in VAT indicate a greater degree of adipose tissue dysfunction. Transcriptional differences in VAT AD comparing SCK vs*.* NK suggest impaired adipogenesis, while SCK VAT IMC exhibited a more pro-inflammatory transcriptional profile, and EC had a more anti-angiogenic profile than non-ketotic cows. In ketotic conditions, SAT and VAT had an increased number of predicted interactions associated with collagen, laminin, and VEGF signaling compared to non-ketotic cows. Although these interactions may be supporting a return to adipose tissue homeostasis in SCK SAT, the increased involvement of immune cells, exhibiting a more pro-inflammatory profile, in these signaling pathway interactions in SCK VAT may be reflective of impaired remodeling or fibrosis.

## Conclusions

With our results, we have provided new information regarding the relationship between SCK and adipose tissue cellular and transcriptional heterogeneity. While SCK appears to induce homeostatic mechanisms to maintain or restore healthy tissue function in SAT, the effects in VAT indicate a greater degree of dysfunction. Moving forward, longitudinal studies across the pre- and postpartum period will be essential for understanding the cellular dynamics of adipose tissue and its impact on the development or resistance to SCK. Furthermore, in vitro studies validating these transcriptional changes at the functional level are necessary and will provide insight into potential molecular targets for improving dairy cattle health and productivity.

## Supplementary Information

Additional file 1: DEGs, GO Terms, REVIGO results for comparisons between AD1, AD2, AD3

Additional file 2: DEGs, GO Terms, REVIGO results for comparisons between ASPC1 and ASPC2

Additional file 3: DEGs, GO Terms, REVIGO results for IMCs

Additional file 4: DEGs, GO Terms, REVIGO results for ECs

Additional file 5: CellChat results for NK SAT, SCK SAT, NK VAT, SCK VAT

## Data Availability

The datasets supporting the conclusions of this article are available in the NCBI GEO repository (Accession #GSE302281).

## Abbreviations

AD: Adipocytes
ASPC: Adipose stem/progenitor cells
BHB: β-Hydroxybutyrate
DIM: Days in milk
EC: Endothelial cells
EndoMT: Endothelial-mesenchymal transitions
GO: Gene Ontology
IMC: Immune cells
LAM: Lipid-associated macrophages
LEC: Lymphatic endothelial cells
MON: Monocytes
NEFA: Non-esterified fatty acids
NK: Non-ketotic
NKT: Natural killer/T-cells
PVM: Perivascular macrophages
SAT: Subcutaneous adipose tissue
SCK: Subclinical ketosis
Sn-RNASeq: Single-nuclei RNA Sequencing
VAT: Visceral adipose tissue
